# A Facile Method for Separating and Enriching Nano and Submicron Particles from Titanium Dioxide Found in Food and Pharmaceutical Products

**DOI:** 10.1371/journal.pone.0164712

**Published:** 2016-10-31

**Authors:** James J. Faust, Kyle Doudrick, Yu Yang, David G. Capco, Paul Westerhoff

**Affiliations:** 1 Molecular and Cellular Biosciences, School of Life Sciences, Arizona State University, Tempe AZ 85287-4501, United States of America; 2 Department of Civil and Environmental Engineering and Earth Sciences, 156 Fitzpatrick Hall, Notre Dame IN, 46556, United States of America; 3 School of Sustainable Engineering and the Built Environment, Arizona State University, Tempe AZ 85287-5306, United States of America; Institute of Materials Science, GERMANY

## Abstract

Recent studies indicate the presence of nano-scale titanium dioxide (TiO_2_) as an additive in human foodstuffs, but a practical protocol to isolate and separate nano-fractions from soluble foodstuffs as a source of material remains elusive. As such, we developed a method for separating the nano and submicron fractions found in commercial-grade TiO_2_ (E171) and E171 extracted from soluble foodstuffs and pharmaceutical products (*e*.*g*., chewing gum, pain reliever, and allergy medicine). Primary particle analysis of commercial-grade E171 indicated that 54% of particles were nano-sized (*i*.*e*., < 100 nm). Isolation and primary particle analysis of five consumer goods intended to be ingested revealed differences in the percent of nano-sized particles from 32%‒58%. Separation and enrichment of nano- and submicron-sized particles from commercial-grade E171 and E171 isolated from foodstuffs and pharmaceuticals was accomplished using rate-zonal centrifugation. Commercial-grade E171 was separated into nano- and submicron-enriched fractions consisting of a nano:submicron fraction of approximately 0.45:1 and 3.2:1, respectively. E171 extracted from gum had nano:submicron fractions of 1.4:1 and 0.19:1 for nano- and submicron-enriched, respectively. We show a difference in particle adhesion to the cell surface, which was found to be dependent on particle size and epithelial orientation. Finally, we provide evidence that E171 particles are not immediately cytotoxic to the Caco-2 human intestinal epithelium model. These data suggest that this separation method is appropriate for studies interested in isolating the nano-sized particle fraction taken directly from consumer products, in order to study separately the effects of nano and submicron particles.

## Introduction

Nanoparticles are becoming increasingly common in food and pharmaceuticals [1]. Whether this is done intentionally or unknowingly as an artifact of commercial-grade material processing [2, 3] is unknown. These additives are mixtures of nano- and submicron-scale particles and are used in consumer goods including foodstuffs as color and texture modifiers [1], moisture controllers [4, 5], or antimicrobials [6]. Indeed, human foodstuffs contain titanium dioxide (TiO_2_) because of its white color and silicon dioxide (SiO_2_) as anticaking agents, and the penetration depth of silver nanoparticle coatings has been tested on fruit presumably because of the antimicrobial properties of silver[7]. At present, a number of definitions for the word nanoparticle exist including size [8], surface area [9], and physicochemical properties that dictate unique behavior [10]. This study adheres to the National Nanotechnology Initiative (NNI) definition, where a nanoparticle has primary particle sizes below 100 nm, though new non-sized based definitions may be on the horizon [10]. Herein, we define particles with sizes above 100 nm to be submicron particles, with the understanding that this definition may change as the field advances.

Recent studies have indicated the presence of TiO_2_ in human foodstuffs [1, 11], signifying the need for characterization of TiO_2_ additives. These studies have shown that TiO_2_ additives have a variety of nano- and submicron-sized particles as determined by passing the extracted material through 0.45 μm filters [1]. Although the intended use of the additive is benign, the fact that portions of these additives are nano-scale raises concern that this subset of nanomaterial in the additive could interact with the human gastrointestinal tract in fundamentally different ways compared to its submicron counterpart [12]. Most recently, TiO_2_ found in pharmaceuticals was found in the bloodstream of human volunteers [13]. Nanoparticles can undergo physicochemical transformations as the material changes environments (*e*.*g*., from foodstuff matrices to the gastrointestinal tract to the bloodstream), and this could lead to unexpected adverse effects [14].

Consequently, there has been a paradigm shift in exposure assessment from employing material purchased as TiO_2_ nanoparticles [15, 16] to employing TiO_2_ that is designated as food-grade (*i*.*e*., E171) or even isolated directly from human foodstuffs [2, 3, 12, 17]. These latter studies focused on the biology of exposure have shown that when particles isolated directly from consumer products are applied to an in vitro model of the human absorptive epithelium they disrupt the number and organization of brush border microvilli [2, 12, 18–22]. Brush border microvilli exist to increase the surface area of the gastrointestinal tract for the purpose of absorption. Deregulation of the absorptive capacity of the gastrointestinal tract can lead to malnutrition or diarrhea. The physical forces associated with sedimentation of food grade TiO_2_ particle agglomerates is not a causal agent for brush border disruption. Furthermore, leaching of ions or reactive species due to the cell culture milieu does not affect brush border microvilli [23]. However, from a physiological standpoint smaller particles more readily pass anatomical barriers and may interact with the surface of the absorptive epithelium in fundamentally different ways compared to bulk counterparts. Thus, if we are to understand if a population of particles taken directly from consumer products exist that affects brush border microvilli it is important to develop protocols that enable separation.

In order to address this problem, a number of analytical tools designed to separate particles based on defined criteria (*e*.*g*., size, charge) have been developed. The “gold-standard” analytical method for particle separation is field flow fractionation (FFF), and this technique has been used in order to characterize the presence of nanoparticles in a number of human foodstuffs [24]. Although FFF is potentially the best way to separate nanoparticles, the method requires equipment that can be costly, may not be readily available, adds contamination to the sample (*e*.*g*., surfactants), and requires technical skill to elute various fractions. An alternative method to separate nano from submicron particles is filtration [24]. The advantages of filters are that they are cost effective and easy to use. However, filter pores are easily obstructed by particles, and collecting the submicron fraction can be technically challenging because the filter cake will contain nanoparticles trapped within the submicron particle matrix. For these reasons, this investigation defines a simple alternative methodology for separating nano and submicron particles from mixtures of TiO_2_ additives intended for human ingestion using equipment common in most laboratories.

The aim of this study was to develop a simple and cost-effective preparative method for separating nano- and submicron-sized particles taken directly from consumer products. TiO_2_ isolated from five soluble consumer products (chewing gum, name-brand allergy medicine, generic allergy medicine, name-brand pain reliever, and generic pain reliever) contained a wide range of nano- and submicron-sized particles. A rate-zonal centrifugation method was used to separate particle sizes on the basis of viscosity gradients. The method separated nano- and submicron-fractions for commercial-grade E171 and E171 isolated from foodstuffs and pharmaceuticals. We then demonstrated the applicability of this method for *in vitro* studies evaluating the nano and submicron effects of E171 isolated from chewing gum on human intestinal cells.

## Materials and Methods

### Titanium Dioxide Isolation

TiO_2_ was isolated from the following soluble consumer products intended for human ingestion: gum that had a hard shell, two pain relievers, and two allergy medicines. TiO_2_ was extracted using an adaptation of a previous method [1]. For gum, 2.5 servings were selected based on the amount of starting material that occupied the 15 mL centrifuge tube and the fact that large quantities of sugars that are components of the candy coating make centrifugation inefficient. The gum was suspended in 10 mL of sterile Nanopure^™^ water with gentle agitation for 10 minutes at room temperature. The liquid containing TiO_2_ (*e*.*g*., gum-E171) was decanted into a separate 15 mL centrifuge tube, and the tube containing the gum base that contained trace amounts of TiO_2_ was discarded according to institutional guidelines. Endotoxin/nuclease-free microcentrifuge tubes (VWR, 16466–030) were filled with 1.5 mL of TiO_2_ isolate and centrifuged with a fixed-angle microcentrifuge rotor (Fischer Scientific, Submicron 14) at 14,000 × g for 10 minutes. This extensive preliminary centrifugation step was necessary because of the sugars that were solubilized from the candy coating. The translucent supernatant containing sugars and devoid of TiO_2_ was decanted, and the volume in the tube was brought to 1.5 mL with sterile Nanopure^™^ water. The pellet containing the TiO_2_ was re-suspended by sonication with a microprobe sonicator (Fisher Scientific, Sonic Dismembrator Model 100) for 10 seconds at maximum output (29 watts RMS). The liquid containing TiO_2_ was subsequently centrifuged at 14,000 × g for 3 minutes to produce a TiO_2_ pellet. The wash liquid was decanted, and the re-suspension step via sonication was repeated. This washing process, starting with re-suspending the TiO_2_ pellet in sterile Nanopure^™^ water and ending with centrifugation at 14,000 × g for 3 minutes, was repeated for a total of 5 times. Immediately following the 5 water washes, the TiO_2_ was washed with non-denatured absolute ethanol via the aforementioned wash procedure for a total of 5 times. After the final pelleting step, the ethanol was decanted and the microcentrifuge tube was incubated in a drying oven at 68°C overnight to thoroughly dry the sample.

Concerning the samples with more complex components (*i*.*e*., over-the-counter drugs in the form of pills); five pills as an arbitrary quantity were added to a 15 mL centrifuge tube containing 10 mL of sterile Nanopure^™^ water and incubated for 1 hour with gentle agitation at 25°C. The liquid slurry was sonicated for 30 seconds at maximum output. The slurry was centrifuged for 3 minutes at 125 × g in order to remove the large debris. The supernatant was collected, and 1.5 mL of supernatant was added to endotoxin/nuclease-free microcentrifuge tubes (VWR, 16466–030). The entire procedure for washing and drying described in the preceding paragraph for the TiO_2_ isolate was conducted.

### Isolation of Nano/Submicron Enriched TiO_2_ Fractions

Ultrapure sucrose (Sigma Aldrich, S7903) solutions (50% m/v and ~70–75% m/v, *i*.*e*., saturated) were made by overnight incubation at room temperature in sterile Nanopure^™^ water. Sucrose step gradients were created by applying 250 μL of saturated sucrose to the bottom of a microcentrifuge tube (VWR, 16466–030) and carefully layering 500 μL of 50% sucrose on top. Immediately before use, dried TiO_2_ pellets were re-suspended in 500 μL of sterile Nanopure^™^ water and sonicated with a microprobe sonicator (Fisher Scientific, Sonic Dismembrator Model 100) for 10 seconds at maximum output (29 watts RMS). The 500 μL of TiO_2_ was carefully layered on top of the 50% sucrose solution, and the entire step gradient was centrifuged at 25°C for 3 minutes at 12,000 × g. After this centrifugation process, the centrifuge tube had a pellet at the bottom and a turbid appearance in all sucrose and water layer(s). The top 2 layers (500 μL water/sample and 500 μL 50% sucrose) were carefully collected by aspirating from the meniscus (1 mL total “nano” volume) and this pooled fraction was placed in a new microcentrifuge tube. This new microcentrifuge tube sample is hereafter referred to as the “nano tube.” The microcentrifuge tube containing the pellet beneath the saturated sucrose was set aside and hereafter referred to as the “submicron tube.” The washing and drying procedures described in the *Titanium Dioxide Isolation* section were conducted in order to obtain a nano-enriched fraction for later use.

The submicron-enriched fraction was procured by gently inverting the submicron tube to decant the saturated sucrose supernatant containing the mixture and gently washing the sides of the inverted submicron tube with sterile Nanopure^™^ water. The pellet was washed and dried according to the procedure described in the *Titanium Dioxide Isolation* section in order to obtain a submicron-enriched fraction for later use.

### Electron Microscopy and Primary Particle Analysis

TiO_2_ isolates were re-suspended in sterile Nanopure^™^ water at a concentration of 10 ppm (*i*.*e*., 10 μg/mL) and drop-casted onto formvar-coated slot grids (Electron Microscopy Sciences, FF-2010-Cu). The grids were dried overnight and imaged with a Philips CM-12 transmission electron microscope (TEM) fitted with a Gatan 791 sidemount CCD at an accelerating voltage of 80 kV. At least 10 images were collected per sample. Images were analyzed with ImageJ [5, 25], and primary particle sizes were calculated by measuring the edge-to-edge particle length and the length normal to this (*i*.*e*., X and Y direction) from each particle in the field of view. The two measurements were averaged for each single particle to obtain an average particle size. The sample primary particle size reported was calculated from 300‒1,000 average particle sizes.

A scanning electron microscope (SEM) fitted with a field emission gun (XL-30; FEI, Oregon, USA) and equipped with energy dispersive x-ray (EDX) microanalysis was used to obtain images of the isolated particles and their composition, respectively. Samples were prepared on conductive carbon tape. At least three EDX analyses were performed for each sample on areas approximately 250–500 nm in width and height.

### Cell Culture and Scanning Electron Microscopy of Caco-2BBe1 Epithelia

The human brush border expressing cell line (Caco-2 BBe1; obtained from ATCC at passage number 47; CRL-2102) was maintained as described elsewhere [2, 12]. Briefly, cells were grown for 19–21 days in order to permit differentiation of the confluent epithelium. The cell culture medium is DMEM (Cellgro, 10-013-CM) supplemented with 10 μg/mL of human transferrin (Invitrogen, 30124SA), 1% antibiotics (Cellgro, 30-004-CI), and 10% fetal bovine serum (Atlanta Biological, S-11150). Preliminary experiments were conducted in order to determine the minimum amount of time required for submicron-enriched TiO_2_ isolate to adhere to the epithelium as determined by scanning electron microscopy. This time point (7 minutes) was used as a baseline for the remainder of the studies. Epithelia were cultured in the inverted position by adding the epithelia growing on a substrate to the bottom of a 15 mL centrifuge tube. Because the epithelium grows only on one side of the substrate, the epithelium can be inverted by tilting the centrifuge tube 90° while monitoring which side the epithelium is facing. The centrifuge tube cap was fastened half way to permit gas exchange and returned to the cell culture incubator immediately after applying medium containing nano or submicron TiO_2_ fractions.

Epithelia were washed once with phosphate-buffered saline (Cellgro, 21–030) in the inverted position and cytologically fixed for 1 hour at 25°C. The primary fixative is composed of the following reagents: 2% electron microscopy grade glutaraldehyde (Electron Microscopy Sciences, 16020), 1% electron microscopy grade formaldehyde (Electron Microscopy Sciences, 15712), 100 mM PBS, pH 7.2. Epithelia were incubated in fixative for no longer than 30 minutes. The epithelia were maintained in the upright position after the primary fixation step. Immediately following primary fixation, the epithelia were washed 10 times for 15 minutes each in copious amount of PBS. The secondary fixative, which was necessary to preserve the membranes in these biological samples, was electron microscopy grade osmium tetroxide (OsO_4_; Electron Microscopy Sciences, 19150). OsO_4_ was diluted in sterile Nanopure^™^ water to a final working concentration of 1%. The secondary fixative was applied to the epithelia immediately after the final PBS wash. The epithelia were post-fixed with 1% OsO_4_ for 45 minutes at 25°C in a well-ventilated chemical hood. Epithelia were washed with copious volumes of sterile Nanopure^™^ water 10 times for 15 minutes each. Dehydration of the specimens, which is necessary to critical point dry the specimens, was accomplished with a graded acetone series. The specimens were dried through the CO_2_ critical point and sputter coated with palladium/gold. Scanning electron micrographs were collected on a JOEL JSM6300. Neither air drying nor drying with solvents circumvented the artifacts associated with surface tension (Figure A in S1 File) indicating that the washing steps necessary to dehydrate and critical point dry the specimen are necessary if surface structures are to be viewed.

### Live-Dead Analysis

Live-Dead analysis was conducted as described previously [26]. Briefly, Caco-2 BBe1 epithelia were exposed to the 3 separate fractions as replicate specimens for 24 hours. Ethidium-homodimer 1 was applied to complete culture medium according to the manufacturer’s recommendation (Thermo Fisher, E1169), and incubated for 30 minutes. Epithelia were subsequently washed with complete culture medium 2 times rapidly and imaged live as previously described [26]. Under these conditions ethidium-positive cells (pseudocolored red) indicate disruption of the nuclear membrane as a marker of cell death, whereas living cells appear phase-dense with bright white boundaries (phase optics) and have no ethidium fluorescence (pseudocolored red). No fewer than 3 fields corresponding to 500 x 750 μm^2^ were collected from 3 independent experiments.

## Results and Discussion

### Isolation and Particle-Size Analysis of TiO2

**Fig 1:**
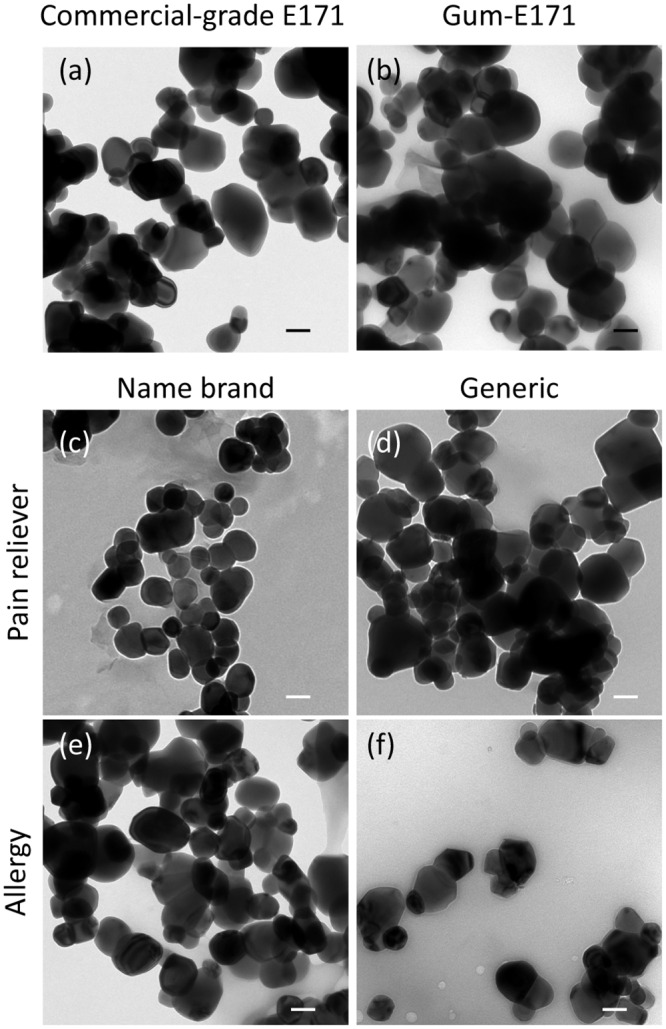
TEM primary particle analysis for TiO_2_ found in selected foodstuffs and pharmaceuticals. (a) commercial-grade E171 (b) chewing gum (c) name-brand allergy medicine, (d) generic allergy medicine, (e) name-brand pain reliever, and (f) generic pain reliever medicine. All images are shown at identical magnification. Scale bar in the lower right corner of each micrograph is 100 nm.

The TiO_2_ primary particle size was determined for commercial-grade E171 (*i*.*e*., E171 before adding to products) and E171 isolated from five water-soluble foodstuff and pharmaceutical products using TEM (Table 1). Commercial-grade E171 contained crystalline (*e*.*g*., tetrahedral) particles as well as a number of particles with amorphous geometries (Fig 1a). Individual particle size averages ranged from 40 to 240 nm. The total average primary particle size was 103 ± 40 nm. Similar to commercial-grade E171, TiO_2_ isolated from the foodstuff and pharmaceutical products was crystalline and contained some particles with amorphous geometries (Fig 1b–1f). The total average primary particle size for gum-E171 (Fig 1b), name-brand allergy E171 (Fig 1c), generic allergy E171 (Fig 1d), name-brand pain reliever E171 (Fig 1e), and generic pain reliever E171 (Fig 1f) was 121 ± 55 nm, 94 ± 25 nm, 99 ± 40 nm, 119 ± 39 nm, and 109 ± 39 nm, respectively (Table 1). For all samples, sizes ranged from approximately 25 to 300 nm and the percent nano-sized particles ranged from approximately 30% to 60% as calculated from TEM particle size averages (*e*.*g*., % less than 100 nm). Interestingly, the generic medicines contained a greater quantity of nano-sized particles compared to their name-brand counterparts (*e*.*g*., 32% and 58% for name-brand and generic allergy medicine, respectively). All of the isolated particles analyzed by SEM-EDX were confirmed to be TiO_2_ (S2 Fig), indicating that no other nano-sized compounds (*i*.*e*., SiO_2_) were present in the isolated samples. These data show that commercial-grade E171 and E171 isolated from foodstuffs and pharmaceutical products are polydispersed, containing mixtures of nano- and submicron-sized particles. These particles may have different size-dependent physico-chemical effects, and they can potentially interact with various systems differently (*e*.*g*., *in vitro* cell models). As such, a method for separating the nano- and submicron-sized fractions was needed.

**Table 1:** Primary particle size analysis using TEM for commercial-grade E171and E171 isolated from selected foodstuffs and pharmaceuticals.

Sample Name	Primary Particle Size	Nano (%)
Commercial-grade E171	103 ± 40 nm	54%
Chewing gum	122 ± 49 nm	35%
Name-brand allergy pill	94 ± 25 nm	32%
Generic allergy pill	99 ± 40 nm	58%
Name-brand pain reliever pill	119 ± 39 nm	36%
Generic pain reliever pill	109 ± 39 nm	49%

### Separation of Nano- and Submicron-Sized Particles from Isolates

**Fig 2:**
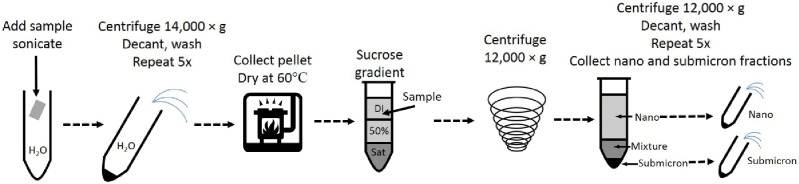
Schematic guide for isolating TiO_2_ (E171) from foodstuffs and pharmaceutical products and separating the nano- and submicron-sized fractions.

Previous studies have characterized E171 [1, 3] and have further investigated the effects of TiO_2_ isolated from consumer goods [12]. However, the individual effects of nano- and submicron-sized particles found in E171 has yet to be determined because of the difficulty in separating the two fractions. Filtration is often employed as a simple method for size separation, but our attempts at isolating the nano- and submicron-sized particles proved inefficient. We used 100 nm and 200 nm membrane filters (cellulose acetate) in an attempt to collect nanoparticles in the filtrate and submicron particles in the filter cake. However, the filter pores were quickly blocked, reducing the number of nanoparticles that passed and increasing the nanoparticle contamination of the filter cake, which agrees with previous results on TiO_2_ nanoparticle filtration [27]. Consequently, a new method was needed. For this study, a rate-zonal sucrose centrifugation separation was developed on the basis of a method frequently used in biological sciences to fractionate subcellular organelles including mitochondria [28], intact brush borders [29], plasma membranes [30], proteins [31], and viruses [32]. Similarly, this separation method is used to purify and enrich specified nanoparticle sizes [33, 34]. The method developed in this study is depicted in Fig 2. TiO_2_ particle size separation was possible using this method because for a sample containing particles with like density (*i*.*e*., only TiO_2_ particles), particle size separation depends on liquid density and viscosity. While the density between 50% sucrose and saturated sucrose does not change significantly (*i*.*e*., 1.231 and 1.381 g/mL at 20°C, respectively[35]), the viscosity changes by two orders of magnitude (*i*.*e*., 15.43 and 2338 cP at 20°C, respectively[36]). During centrifugation, smaller particles were slowed by the difference in liquid density and viscosity over the short term, whereas larger particles were less affected by viscosity changes in the liquid medium. This resulted in larger particles ending up in the centrifuge pellet and smaller particles staying near the top in the supernatant.

**Fig 3:**
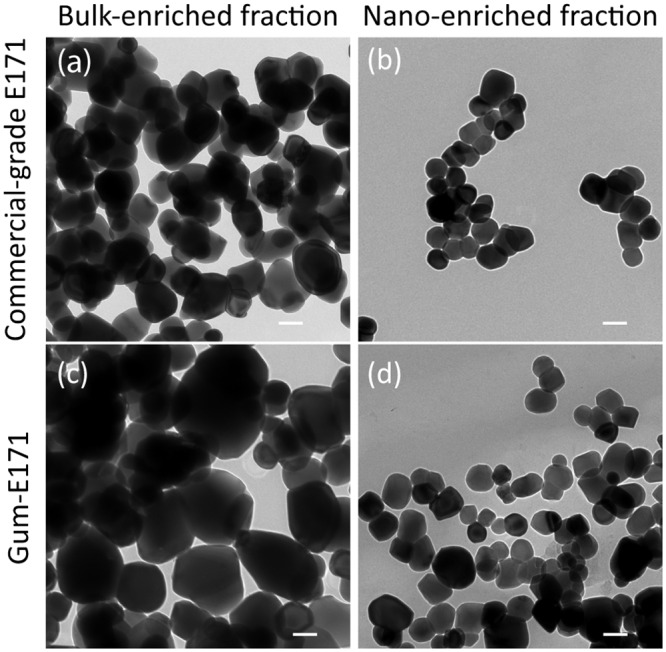
Primary particle analysis via TEM reveals a difference in size between nano- and submicron-enriched fractions after the sucrose step-gradient centrifugation procedure. (a) Submicron-enriched commercial-grade E171 appear large compared to (b) the nano-enriched fraction. (c) Utilizing the same procedure on gum-E171 revealed a large, submicron-enriched fraction and (d) a small, nano-enriched fraction. All images are shown at identical magnification. The scale bar in the lower right corner of each micrograph is 100 nm.

This method was demonstrated for commercial-grade E171 and E171 extracted from gum (gum-E171). Fig 3 shows TEM micrographs of the nano- and submicron-enriched fractions isolated from commercial-grade E171 (Fig 3a and 3b) and gum-E171 (Fig 3c and 3d). The images show a clear size difference between the fractions. The average primary particle size for commercial-grade E171 nano- and submicron-enriched fractions was 77 ± 22 nm (76% nano) and 122 ± 22 nm (31% nano), respectively. The average primary particle size for gum-E171 nano- and submicron-enriched fractions was 95 ± 28 nm (59% nano) and 146 ± 48 nm (16% nano), respectively (Table 2). The gum-E171 sample had the largest overall mean size of all samples (*i*.*e*., 121 ± 55 nm), which is consistent with the large mean size observed for the nano-enriched fraction.

**Table 2:** Primary particle analysis using TEM for commercial-grade E171 and gum-E171 nano- and submicron-enriched fractions.

Sample	Primary Particle Size (nm)	Nano (%)	Submicron (%)	d_10_ (nm)	d_90_ (nm)
Commercial-grade E171 submicron	122 ± 22 nm	31	69	78	180
Commercial-grade E171 nano	77 ± 22 nm	87	13	54	116
Gum-E171 submicron	146 ± 48 nm	15	85	91	208
Gum-E171 nano	95 ± 28 nm	60	40	65	132

**Fig 4:**
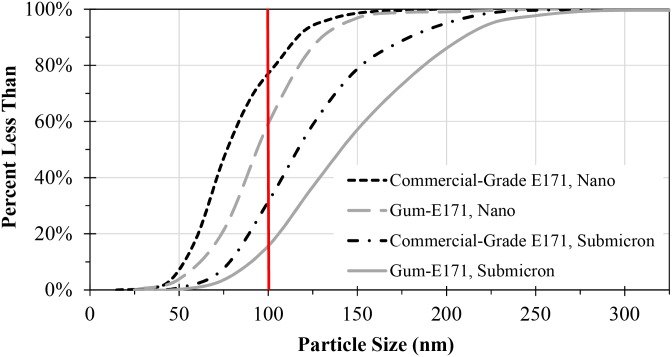
Cumulative size distribution for TEM primary particle analysis of commercial-grade E171 and gum-E171 nano- and submicron-enriched fractions. Solid vertical line indicates nanoparticle size cutoff using the 100 nm definition.

Fig 4 shows the cumulative size distribution for TEM primary particle analysis of the commercial-grade E171 and gum-E171 nano- and submicron-enriched fractions. The cumulative size distribution is useful for determining the percent of particles smaller than a specified size. For example, for the nano-fraction of the gum, 60% of the particles measured by TEM were less than 100 nm. Two other important particle size values are the d_10_ and d_90_ sizes, which represent the lower and upper boundaries of the distribution by removing extreme sizes. The d_10_ sizes (*i*.*e*., 10% of the particles sizes are less than this number) for commercial-grade E171 nano/submicron and gum nano/submicron fractions were 54/78 nm and 65/91 nm, respectively (Table 2). The d_90_ (*i*.*e*., 90% of the particles sizes are less than this number) values for nano and submicron for commercial-grade E171 nano/submicron fractions and gum nano/submicron fractions were 116/180 nm and 132/208 nm, respectively (Table 2). The d_10_ values indicate that for commercial-grade E171 and gum-E171 submicron fractions, the smallest particles were still below 100 nm. The d_90_ values for the nano-fractions of commercial-grade E171 and gum-E171 indicate that submicron particles still remained in the nano-enriched fraction, though the sizes were close to 100 nm.

### Application of Separation Method: Adhesion of nano- and submicron-enriched TiO2 fractions using the Caco-2BBe1 human intestinal and assessment of cell death

**Fig 5:**
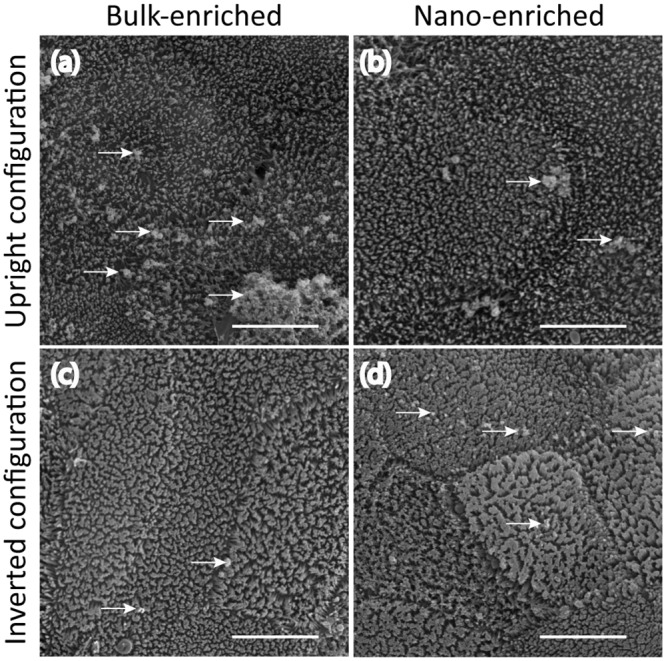
SEM micrographs of human intestine *in vitro* cell models exposed to gum-E171 nano- or submicron-enriched fractions. (a) Epithelia exposed to 1 μg/mL of submicron-enriched fraction in the upright configuration resulted in a large number of particles (white arrows) decorating the surface of the epithelium after 7 minutes of exposure. (b) However, exposing replicate samples to the nano-enriched fraction with 1 μg/mL for 7 minutes in the upright configuration resulted in few particles adhered to the epithelial surface. (c) Inverting the epithelium and subsequently exposing the cells with 1 μg/mL of the submicron-enriched fraction for 7 minutes resulted in few particles adhered to the epithelial surface. (d) However, exposing replicate samples in the inverted configuration to 1 μg/mL of the nano-enriched fraction for 7 minutes resulted in relatively more particles adhered to the epithelial surface. All images are shown at identical magnification. Scale bar is 5 μm.

When performing *in vitro* intestinal cell model studies using E171, determining if the observed effects are due to nano or submicron particles is difficult because TiO_2_ isolated directly from foodstuffs are polydispersed particle-size mixtures [1, 12]. We used the separation method developed in this study to monitor the time necessary for the nano- and submicron-enriched fractions to adhere to the surface of cells grown as epithelia. Because particle settling has been shown to depend on the orientation of the epithelium as well as particle size [12, 37], samples were exposed separately to replicate epithelia in two alternate orientations, upright and inverted, to determine the effect of particle settling. Fig 5 shows SEM images of epithelia exposed separately to gum-E171 nano- and submicron-enriched fractions in inverted and upright configurations. In the upright configuration, submicron-enriched particles adhered to the surface of the epithelia after 7 minutes of exposure (Fig 5a). The micrographs show regions decorated with particles (pointed to with white arrows). However, exposure to the nano-enriched gum-E171 as a parallel replicate resulted in fewer particles adhered to the cell surface (Fig 5b).

The alternative epithelial orientation (*i*.*e*., inverted) has been employed in order to mitigate the effects of sedimentation [12, 37]. In order to investigate the contribution of gum-E171 settling and subsequently adhering to the cell surface, the epithelia were inverted and exposed to gum-E171 nano- and submicron-enriched fractions as replicate samples for 7 minutes. Under these conditions, apparently few particles from the submicron-enriched fraction adhered to the cell surface (Fig 5c). However, when parallel samples were exposed to the nano-enriched fraction, the surface appeared to contain many more nanoparticles and nanoparticle agglomerates adhered to the cells (pointed to with white arrows; Fig 5d). These data suggest that nano- and submicron particles may interact differently with cells over the short term since agglomeration leading to settling is apparently more pronounced for larger TiO_2_ agglomerates than smaller TiO_2_ agglomerates.

This study showed a greater concentration of submicron-enriched particles decorating the surface of the epithelium in the upright, but not the inverted configuration, after as few as 7 minutes of exposure (Fig 5a and 5c). Exactly the opposite trend was observed for epithelia exposed to the nano-enriched fraction (Fig 5b and 5d). Previous studies have shown that the density of the material can drastically affect the actual concentration exposed to the cells [37]. Further, recent studies have shed light on a non-lethal, but significant, effect of exposure to E171 or supplements containing a variety of nanoparticles in a human *in vitro* model of the intestinal epithelium [2, 5, 12]. That is, exposure to E171 resulted in a loss of microvilli from the surface of the cells in both the upright and inverted configuration and during conditions of microgravity [12]. This suggested that the loss of microvilli was not absolutely dependent on sedimentation of the material.

**Fig 6:**
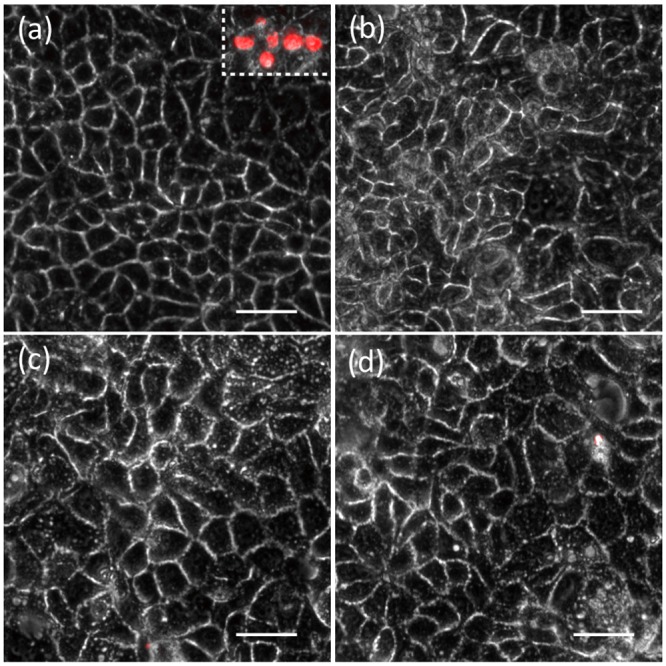
Live-dead analysis indicates that E171 is not immediately cytotoxic to Caco-2 epithelia. (a) Untreated Caco-2 display the traditional honeycomb arrangement of cells within the epithelium. Note the existence of no ethidium-positive (red) cells. The inset is a positive control for cell death. Red cells indicate cell death. (b) Exposure to 1 μg/mL of E171 (total fraction) after 24 hours. (c) Exposure to 1 μg/mL of submicron-enriched E171 after 24 hours. (d) Exposure to 1 μg/mL of submicron-enriched E171 after 24 hours. The scale bars are 20 μm.

Numerous studies have shown that TiO_2_ NPs are not immediately toxic to Caco-2 epithelia [15, 38, 39]. In this study we extend these observations to include E171 size fractions (Fig 6). Under normal, untreated conditions Caco-2 cells maintain the traditional “honeycomb” arrangement when viewed with phase optics, no ethidium-positive cells are observed (Fig 6a). However, treating Caco-2 epithelia with low concentrations of digitonin, used as a positive control for cell death [26] indicates an increase in ethidium-positive cells (Fig 6a inset). Exposure to 1 μg/mL of each of the E171 fractions for 24 hours resulted in apparently no ethidium-positive cells (Fig 6b–6d), suggesting that food-grade particles do not rapidly kill Caco-2 epithelium. Collectively, these data suggest that this method offers a simple tool that can be used in order to identify changes in physico-chemical parameters or toxicity assessment from TiO_2_ particles isolated directly from several consumer goods.

## Conclusions

The major finding in the present investigation was that it was possible to separate submicron particles (>100 nm) from nanoparticles (≤100 nm) isolated directly from foodstuffs and pharmaceuticals containing polydispersed TiO_2_ to produce nano- and submicron-enriched fractions for characterization and toxicity testing. This was first demonstrated for commercial-grade E171 and subsequently confirmed for E171 isolated from water-soluble foodstuffs and pharmaceuticals. Although a number of techniques exist to separate nano and submicron components from consumer goods and foodstuffs [40, 41], many of these techniques are contingent upon equipment that may not be readily available or require the addition of compounds (*e*.*g*., surfactants in FFF) that render the separated particles useless for toxicity tests. Therefore, this study used equipment and reagents that are readily available in most scientific laboratories and safe for toxicity testing. These data further show the relative distribution of size of TiO_2_ particles taken directly from five locally purchased consumer products. We provide evidence that although E171 particles are not immediately toxic in the convention sense, subsequent investigations should determine if brush border disruption is dependent on a particular physicochemical parameter of food grade TiO_2_. Future work should include the development of new extraction methods for separating nanoparticles from non-soluble foodstuffs, and begin mechanistic studies that identify physicochemical parameters that give rise to brush border disruption.

## Supporting Information

S1 File Figure A. SEM images of various drying methods for Caco-2 BBe1. Critical point dried, but not solvent or air dried, brush borders is appropriate for analysis of particle adhesion and brush border disruption. (a) The normal organization of the brush border is observed when Caco-2 BBe1 epithelia are critical point dried. (b) Acetone dried samples result in aggregation of surface microvilli and do not permit analysis of particle adhesion. (c) Air drying the samples results in flattened surface structures as an artifact and does not permit analysis of surface adhesion. All images are shown at identical magnification. Scale bar is 5 μm. Figure B. SEM and EDX showing TiO_2_ sample content for (a) carbon tape (control), (b) gum-E171, (c) pain reliever medicine isolate, and (d) allergy medicine isolate. Scale bar is 250 nm. All images are shown at identical magnification. White box indicates the EDX area being analyzed.
(DOCX)
