# Supplementary material for: A Facile Method for Separating and Enriching Nano and Submicron Particles from Titanium Dioxide Found in Food and Pharmaceutical Products
Source: PLoS One. 2016 Oct 31;11(10):e0164712. doi: 10.1371/journal.pone.0164712 (PMC5087857; doi:10.1371/journal.pone.0164712)
Supplement: S1 File — Figure A. SEM images of various drying methods for Caco-2 BBe1. Critical point dried, but not solvent or air dried, brush borders is appropriate for analysis of particle adhesion and brush border disruption. (a) The normal organization of the brush border is observed when Caco-2 BBe1 epithelia are critical point dried. (b) Acetone dried samples result in aggregation of surface microvilli and do not permit analysis of particle adhesion. (c) Air drying the samples results in flattened surface structures as an artifact and does not permit analysis of surface adhesion. All images are shown at identical magnification. Scale bar is 5 μm. Figure B. SEM and EDX showing TiO2 sample content for (a) carbon tape (control), (b) gum-E171, (c) pain reliever medicine isolate, and (d) allergy medicine isolate. Scale bar is 250 nm. All images are shown at identical magnification. White box indicates the EDX area being analyzed. (DOCX) [file pone.0164712.s001.docx]

**Supporting Information**


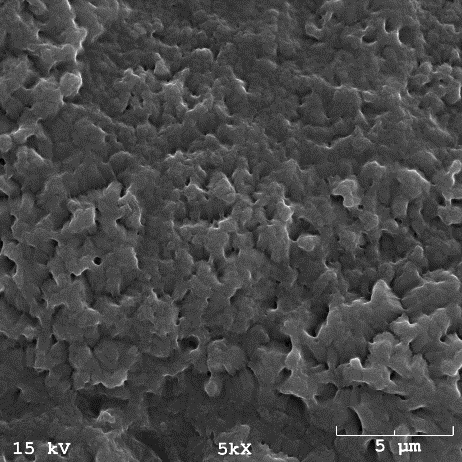

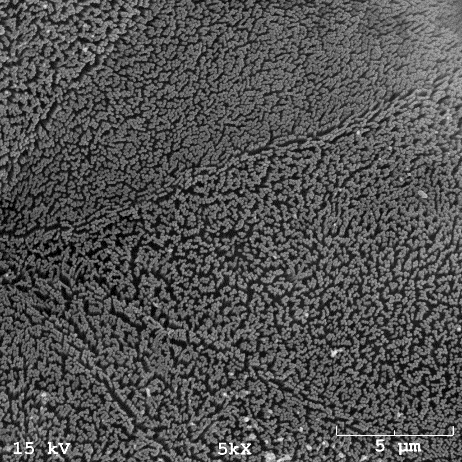

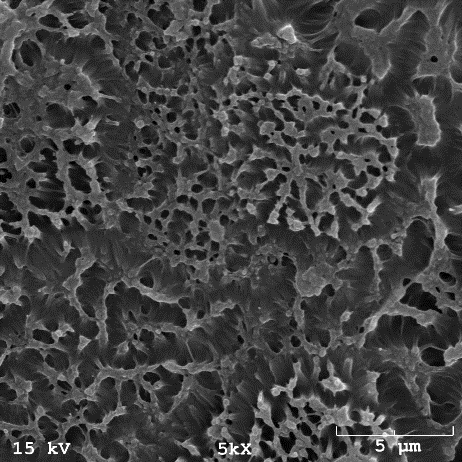


Critical point dried

Acetone dried

Air dried

(a)

(b)

(c)

**Fig SI-1. SEM images of various drying methods for Caco-2 BBe1. Critical point dried, but not solvent or air dried, brush borders is appropriate for analysis of particle adhesion and brush border disruption.** (a) The normal organization of the brush border is observed when Caco-2 BBe1 epithelia are critical point dried. (b) Acetone dried samples result in aggregation of surface microvilli and do not permit analysis of particle adhesion. (c) Air drying the samples results in flattened surface structures as an artifact and does not permit analysis of surface adhesion. All images are shown at identical magnification. Scale bar is 5 µm.


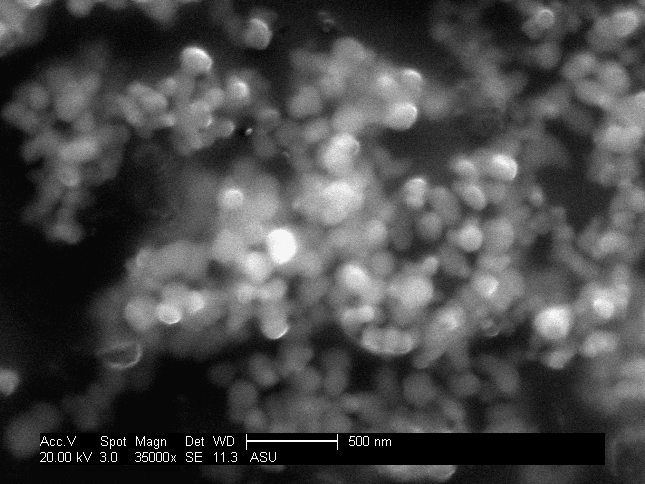

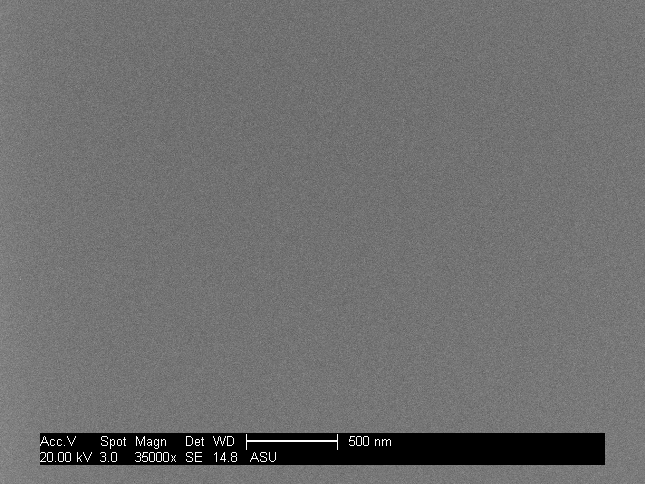


(a)

Carbon Tape

SEM

EDX


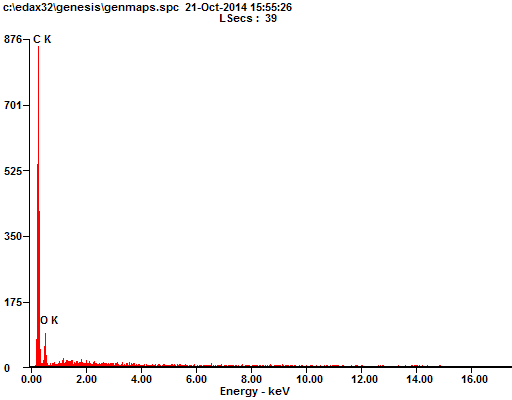

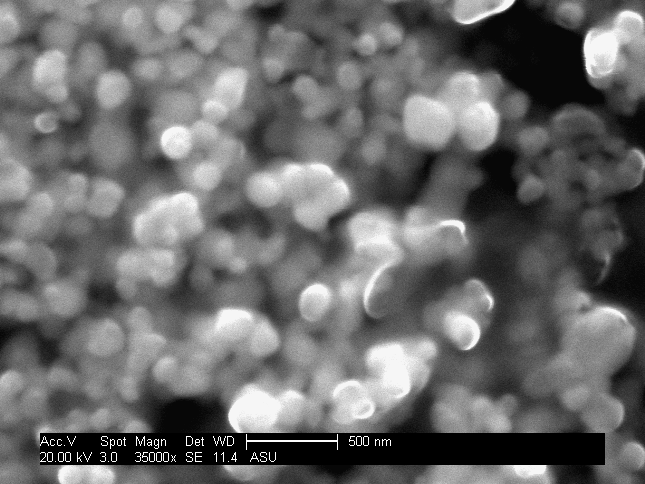

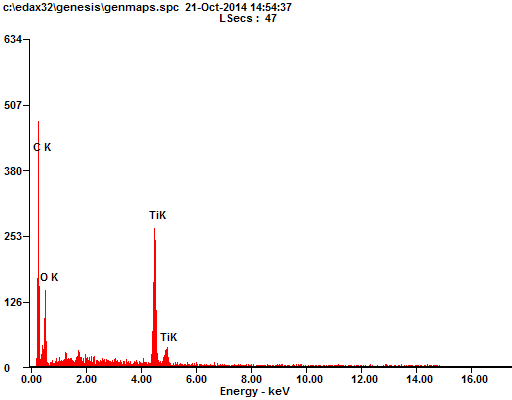


Gum-E171


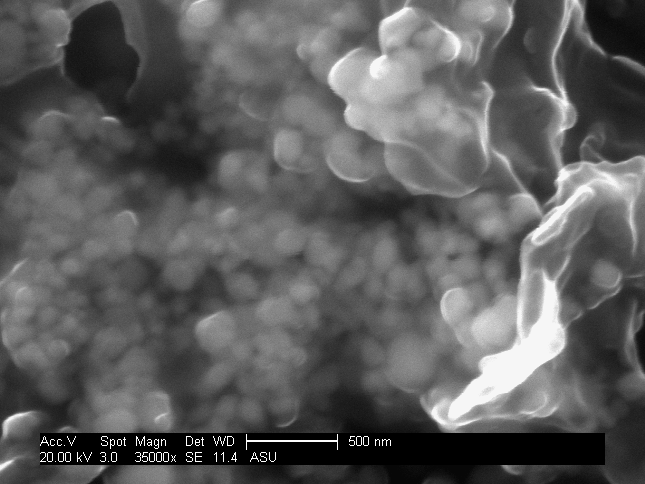


Pain Reliever

Allergy


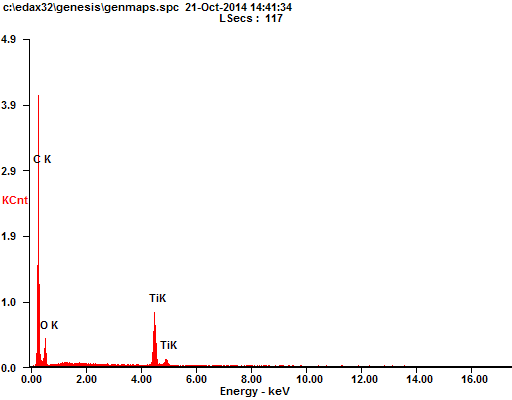

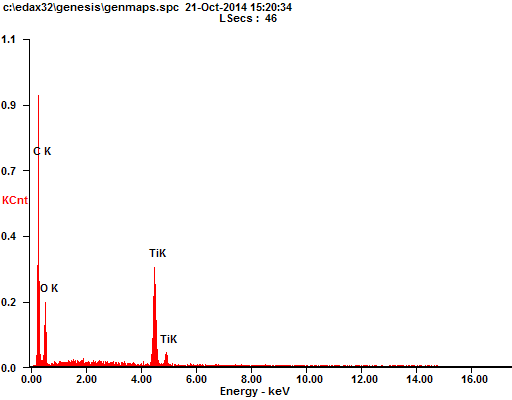


**Fig SI-2. SEM and EDX showing TiO_2_ sample content** for (a) carbon tape (control), (b) gum-E171, (c) pain reliever medicine isolate, and (d) allergy medicine isolate. Scale bar is 250 nm. All images are shown at identical magnification. White box indicates the EDX area being analyzed.
